# A Case Report of Autoimmune Gastritis Associated With Polyendocrine Syndrome Type III Mimicking Refractory H. pylori Infection

**DOI:** 10.7759/cureus.92659

**Published:** 2025-09-18

**Authors:** Saki Ubukata, Hideki Mori, Tatsuhiro Masaoka, Juntaro Matsuzaki, Takanori Kanai

**Affiliations:** 1 Division of Gastroenterology and Hepatology, Department of Internal Medicine, Keio University School of Medicine, Tokyo, JPN

**Keywords:** autoimmune gastritis, graves' disease, helicobacter pylori, type 1 diabetes mellitus, type 3 autoimmune polyendocrine syndrome

## Abstract

A 60-year-old woman had persistent positive urea breath test (UBT) results despite three courses of *Helicobacter pylori* (*H. pylori*) eradication therapy. Endoscopy revealed extensive atrophic gastritis with a characteristic "reverse atrophic pattern" affecting the gastric corpus while sparing the antrum. Active *H. pylori* infection was excluded; however, positivity for anti-parietal cell and anti-intrinsic factor antibodies confirmed autoimmune gastritis (AIG). Further screening identified anti-glutamic acid decarboxylase antibodies and thyroid autoantibodies, leading to a diagnosis of autoimmune polyendocrine syndrome type III. This case highlights the potential for false-positive UBT results in AIG, emphasizing the importance of screening for associated autoimmune conditions.

## Introduction

Autoimmune gastritis (AIG) is a chronic condition characterized by immune-mediated destruction of gastric parietal cells, resulting in atrophic gastritis, achlorhydria, and hypergastrinemia [1,2]. AIG is not only a risk factor for gastric cancer, but is also commonly associated with other autoimmune diseases such as autoimmune thyroid disease and type 1 diabetes mellitus (T1DM) [3,4]. These associations underscore the importance of systematic screening at the time of AIG diagnosis. Early identification of the comorbidities can lead to timely interventions and prevent complications. AIG is often misdiagnosed as refractory *Helicobacter pylori* (*H. pylori*) infection, mainly because the two conditions share overlapping clinical and endoscopic features such as gastric atrophy. This is thought to be due to extensive gastric mucosal atrophy associated with AIG, which may lead to false-positive urea breath test (UBT) results, creating diagnostic confusion [4]. This report presents the case of a female patient referred to us with refractory *H. pylori* infection. Ultimately, she was diagnosed with AIG and autoimmune polyendocrine syndrome type III. This case highlights two critical points: the potential for AIG to be misdiagnosed as a refractory *H. pylori *infection, and the importance of screening for thyroid disease and T1DM in patients with AIG.

## Case presentation

A 60-year-old woman was referred to our department of gastroenterology for evaluation of persistent positive UBT results despite three sequential courses of *H. pylori* eradication therapy. At the previous hospital, both the diagnosis of *H. pylori* infection and the evaluation of eradication were based solely on UBT, and no other tests were performed. The patient remained completely asymptomatic, reporting no dyspepsia, epigastric pain, nausea, or other gastrointestinal symptoms. The patient had a prior diagnosis of Graves’ disease at age 15. At that time, although short-term pharmacological treatment was administered, no routine follow-up was conducted. There was no family history of autoimmune disorders, diabetes mellitus, or gastric cancer. She took no regular medications and had no known drug allergies.

**Figure 1:**
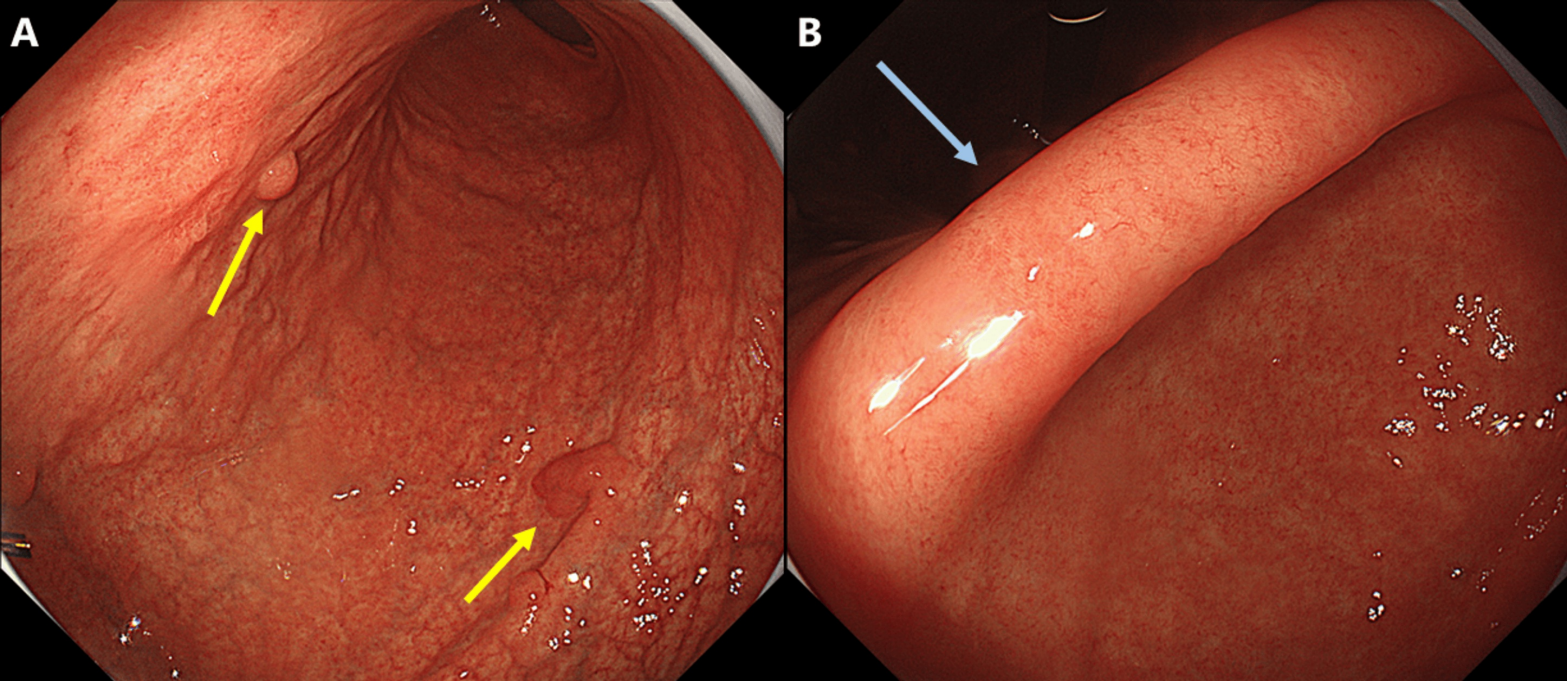
Endoscopic findings. (A) The gastric corpus exhibits marked atrophic changes, with multiple hyperplastic polyps (yellow arrows). (B) Atrophic changes in the antrum are minimal, and a regular arrangement of collecting venules (RAC pattern) is evident in the gastric angle. The RAC pattern is indicated by a blue arrow.

Screening esophagogastroduodenoscopy (EGD) revealed multiple hyperplastic gastric polyps and extensive atrophic gastritis, classified as O-p according to the Kimura-Takemoto classification [5]. Notably, atrophic changes were more pronounced in the gastric corpus, with only minimal atrophy of the antrum (Figure 1).

Histopathological examination of the gastric corpus revealed atrophic changes, inflammatory cell infiltration, and pseudo-pyloric gland metaplasia; however, we found no reduction in parietal cell density or hyperplasia of enterochromaffin-like cells. There was no evidence of *H. pylori* infection on either gastric biopsy culture or gastric fluid polymerase chain reaction (PCR) tests. A repeat UBT performed at our hospital showed a positive result of 5.3‰, exceeding the reference cut-off of 2.5‰ [6]. By contrast, the stool antigen test for *H. pylori* was negative. Taken together, these findings are not consistent with active *H. pylori* infection; therefore, we deemed the UBT result to be a false positive.

We suspected AIG based on the characteristic "reverse atrophic pattern" observed on endoscopy, with pronounced atrophy of the corpus and minimal involvement of the antrum [4]. She was positive for both anti-parietal cell and anti-intrinsic factor antibodies, confirming the diagnosis (Table 1) [7]. Serum gastrin levels were elevated markedly (1770 pmol/L), exceeding the reference cut-off value of 46.9 pmol/L, supporting the diagnosis of AIG.

**Table 1:** Laboratory results. Bold values indicate abnormal results (outside the reference range). WBC: white blood cells; Hb: hemoglobin; Plt: platelets; TP: total protein; Alb: albumin; T-Bil: total bilirubin; AST: aspartate aminotransferase; ALT: alanine aminotransferase; ALP: alkaline phosphatase; γ-GTP: γ-glutamyl transpeptidase; LDH: lactate dehydrogenase; BUN: blood urea nitrogen; Cre: creatinine; Amy: amylase; Na: sodium; K: potassium; Cl: chloride; HbA1c: glycated hemoglobin A1c; Fe: serum iron; TIBC: total iron-binding capacity; UIBC: unsaturated iron-binding capacity; V-B12: vitamin B12; CRP: C-reactive protein; TSH: thyroid-stimulating hormone; FT3: free triiodothyronine; FT4: free thyroxine; TRAb: TSH receptor antibody; TgAb: anti-thyroglobulin antibody; TPOAb: anti-thyroid peroxidase antibody; ACTH: adrenocorticotropic hormone; APCA: anti-parietal cell antibody; AIFAs: anti-intrinsic factor antibodies; GADAb: anti-glutamic acid decarboxylase antibody; CEA: carcinoembryonic antigen; CA19-9: carbohydrate antigen 19-9; NSE: neuron-specific enolase.

Variable	Result	Reference range	Variable	Result	Reference range
WBC (/μL)	5000	3,300-8,600	Fe (μg/dL)	149	40-188
RBC (x10^6^/μL)	5.15	3.86-4.92	TIBC (μg/dL)	448	246-410
Hb (g/dL)	14.1	11.6-14.8	UIBC (μg/dL)	375	180-270
Ht (%)	43.3	35.1-44.4	V-B12 (pg/mL)	196	233-914
MCV (fL)	84.1	83.6-98.2	Ferritin (ng/mL)	8	5-179
Plt (x10^4^/μL)	30.5	15.8-34.8	CRP (mg/dL)	0.07	0-0.14
TP (g/dL)	7.3	6.6-8.1	TSH (μIU/mL)	3.88	0.61-4.23
Alb (g/dL)	4.1	4.1-5.1	FT3 (pg/mL)	2.5	2-4.5
T-Bil (mg/dL)	0.9	0.4-1.5	FT4 (ng/dL)	1.3	0.7-1.8
AST (IU/L)	25	13-30	TRAb (IU/L)	4.2	0-1.9
ALT (IU/L)	18	7-23	TgAb (IU/mL)	33.7	0-4.9
ALP (IU/L)	91	38-113	TPOAb (IU/mL)	932.7	0-2.9
γ-GTP (IU/L)	32	9-32	ACTH (pg/mL)	27.4	8.7-61.5
LDH (IU/L)	203	124-222	Cortisol (μg/dL)	16.6	4.4-21.1
BUN (mg/dL)	16.8	8-20	APCA	1:80	
Cre (mg/dL)	0.67	0.46-0.79	AIFAs	positive	
Amy (IU/L)	149	44-132	GADAb (IU/mL)	2000	0-4.9
Na (mEq/L)	141.3	138-145	CEA (ng/mL)	1.0	0-5
K (mEq/L)	4.6	3.6-4.8	CA19-9 (U/mL)	9	0-37
Cl (mEq/L)	102	101-108	NSE (ng/mL)	15.9	0-16.3
Glucose (mg/dL)	82	73-109	Gastrin (pmol/L)	1770	11.9-46.9
HbA1c (%)	5.9	4.9-5.9			

Considering the diagnosis, we performed further evaluations to screen for disorders known to be associated with AIG, such as iron deficiency anemia, pernicious anemia, thyroid diseases, and T1DM (Table 1). Although hemoglobin and serum iron levels were within normal ranges, serum ferritin was at the lower limit of normal, and total iron-binding capacity was elevated, suggesting a pre-latent stage of iron deficiency. Vitamin B12 levels were slightly low at 196 pg/mL, indicating early-stage deficiency. Blood glucose and HbA1c levels were within normal limits; however, anti-glutamic acid decarboxylase (GAD) antibodies were strongly positive, leading to a diagnosis of latent autoimmune diabetes. Thyroid function tests revealed positivity for anti-thyroglobulin antibodies, anti-thyroid peroxidase antibodies, and thyroid-stimulating hormone receptor antibodies, whereas thyroid-stimulating hormone, free T3, and free T4 were within normal ranges. These findings were consistent with latent forms of both Graves’ disease and Hashimoto’s thyroiditis (Table 1).

The constellation of positive anti-GAD antibodies, thyroid autoantibodies, and AIG led to a diagnosis of autoimmune polyendocrine syndrome type III (APS-III) [8]. The patient was referred to the endocrinologist for ongoing follow-up of latent autoimmune diabetes, as well as the latent forms of both Graves’ disease and Hashimoto’s thyroiditis. In parallel, she was scheduled for regular follow-up in the department of gastroenterology, including annual endoscopic examinations and periodic monitoring of serum vitamin B12 and iron levels.

## Discussion

This case illustrates a unique clinical scenario in which persistent false-positive UBT results led to an incidental diagnosis of AIG and APS-III. The patient’s presentation highlights several important diagnostic considerations and clinical pearls relevant to practice of internal medicine. The most intriguing aspect of this case is the persistent positive UBT despite comprehensive negative *H. pylori* test results. Although UBT, along with the stool antigen test, is regarded as the gold standard for confirming *H. pylori* infection, false-positive results can occur under specific clinical conditions [9-11]. In patients with AIG, the mechanism underlying a false-positive UBT likely involves bacterial overgrowth (mainly *Proteus mirabilis*, *Citrobacter freundii*, *Klebsiella pneumoniae*, *Enterobacter cloacae*, and certain *Streptococcus* species) in the hypochlorhydria stomach environment, which can metabolize urea and produce a positive UBT result [12,13]. AIG is an organ-specific autoimmune disorder characterized by immune-mediated destruction of gastric parietal cells, leading to achlorhydria, hypergastrinemia, and eventually pernicious anemia [1,2]. Diagnosis relies on a combination of clinical, serological, endoscopic, and histopathological findings. Our patient demonstrated classic features such as "reverse atrophic pattern" on endoscopy, in which atrophy predominantly affects the gastric body and fundus while sparing the antrum; this pattern is opposite to that typically seen in cases of *H. pylori*-associated gastritis. For patients exhibiting "reverse atrophic pattern", if the urea breath test is positive, a stool antigen test should also be performed.

The simultaneous presence of thyroid autoimmunity and positive anti-GAD antibodies in our patient is suggestive of APS-III, which is characterized by autoimmune thyroid disease combined with other organ-specific autoimmune conditions, excluding adrenal insufficiency. APS-III is subdivided into three variants: APS-IIIa (thyroid disease + type 1 diabetes), APS-IIIb (thyroid disease + pernicious anemia/AIG), and APS-IIIc (thyroid disease + other autoimmune conditions) [8]. Given the presence of anti-GAD antibodies, our patient exhibits features of both the APS-IIIa and APS-IIIb variants, suggesting latent autoimmune diabetes in adults; therefore, she was confirmed as having AIG. The coexistence of thyroid disease and AIG has been well-documented in the literature, with evidence suggesting overlapping genetic predispositions, including associations with specific human leukocyte antigen (HLA) haplotypes and familial clustering [14-16]. Cases fulfilling criteria for both APS-IIIa and IIIb have been reported, including patients with type 1 diabetes and autoimmune thyroiditis who subsequently developed autoimmune gastritis or pernicious anemia. These overlaps likely reflect shared genetic susceptibility and the natural history from latent autoimmunity to clinical disease, supporting proactive surveillance of vitamin B12/iron status and gastric pathology in APS-III [17].

A diagnosis of AIG carries significant clinical implications beyond gastrointestinal manifestations. Patients with AIG have an increased risk of gastric adenocarcinoma and neuroendocrine tumors, necessitating regular endoscopic surveillance [18,19]. Our patient has multiple hyperplastic polyps, which will require ongoing surveillance as these lesions, while typically benign, can occasionally harbor dysplastic changes.

This case highlights several important diagnostic challenges. First, clinicians should maintain a high index of suspicion for alternative diagnoses in cases where the UBT remains positive despite multiple eradication attempts. Second, a diagnosis of AIG should prompt a comprehensive evaluation for potential comorbidities and related autoimmune conditions. Although the patient had not yet developed anemia, reduced levels of iron and vitamin B12 were observed, indicating the need for continued monitoring. In this case, screening enabled us to identify previously unrecognized latent autoimmune diabetes and autoimmune thyroid diseases, allowing for the possibility of early intervention should these conditions progress to clinical onset.

## Conclusions

In conclusion, this case highlights how a persistent false-positive UBT result led to the unexpected diagnosis of AIG and APS-III, underscoring the importance of comprehensive evaluation and awareness of autoimmune disease associations in the setting of repeated failure of *H. pylori* eradication therapy.

## References

[1] Kulnigg-Dabsch S (2016). Autoimmune gastritis. Wien Med Wochenschr.

[2] Lenti MV, Rugge M, Lahner E (2020). Autoimmune gastritis. Nat Rev Dis Primers.

[3] Song M, Latorre G, Ivanovic-Zuvic D, Camargo MC, Rabkin CS (2019). Autoimmune diseases and gastric cancer risk: a systematic review and meta-analysis. Cancer Res Treat.

[4] Kamada T, Maruyama Y, Monobe Y, Haruma K (2022). Endoscopic features and clinical importance of autoimmune gastritis. Dig Endosc.

[5] Nakajima S, Watanabe H, Shimbo T (2021). Incisura angularis belongs to fundic or transitional gland regions in Helicobacter pylori-naïve normal stomach: sub-analysis of the prospective multi-center study. Dig Endosc.

[6] Ohara S, Kato M, Asaka M, Toyota T (1998). Studies of 13C-urea breath test for diagnosis of Helicobacter pylori infection in Japan. J Gastroenterol.

[7] Kamada T, Watanabe H, Furuta T (2023). Diagnostic criteria and endoscopic and histological findings of autoimmune gastritis in Japan. J Gastroenterol.

[8] Betterle C, Furmaniak J, Sabbadin C, Scaroni C, Presotto F (2023). Type 3 autoimmune polyglandular syndrome (APS-3) or type 3 multiple autoimmune syndrome (MAS-3): an expanding galaxy. J Endocrinol Invest.

[9] Sabbagh P, Mohammadnia-Afrouzi M, Javanian M (2019). Diagnostic methods for Helicobacter pylori infection: ideals, options, and limitations. Eur J Clin Microbiol Infect Dis.

[10] Mori H, Suzuki H, Matsuzaki J, Masaoka T, Kanai T (2020). 10-year trends in Helicobacter pylori eradication rates by sitafloxacin-based third-line rescue therapy. Digestion.

[11] Mori H, Suzuki H (2020). Update on quinolone-containing rescue therapies for Helicobacter pylori infection. World J Gastroenterol.

[12] Osaki T, Mabe K, Hanawa T, Kamiya S (2008). Urease-positive bacteria in the stomach induce a false-positive reaction in a urea breath test for diagnosis of Helicobacter pylori infection. J Med Microbiol.

[13] Furuta T, Baba S, Yamade M (2018). High incidence of autoimmune gastritis in patients misdiagnosed with two or more failures of H. pylori eradication. Aliment Pharmacol Ther.

[14] Whittingham S, Youngchaiyud U, Mackay IR, Buckley JD, Morris PJ (1975). Thyrogastric autoimmune disease. Studies on the cell-mediated immune system and histocompatibility antigens. Clin Exp Immunol.

[15] Baxter AG, Jordan MA, Silveira PA, Wilson WE, Van Driel IR (2005). Genetic control of susceptibility to autoimmune gastritis. Int Rev Immunol.

[16] Levin L, Ban Y, Concepcion E, Davies TF, Greenberg DA, Tomer Y (2004). Analysis of HLA genes in families with autoimmune diabetes and thyroiditis. Hum Immunol.

[17] Subtil J, Carvalho R, Rebelo AF, Subtil P (2024). Autoimmune polyglandular syndrome type 3: a case report. Cureus.

[18] Rustgi SD, Bijlani P, Shah SC (2021). Autoimmune gastritis, with or without pernicious anemia: epidemiology, risk factors, and clinical management. Therap Adv Gastroenterol.

[19] Zhang T, Tang X (2025). Beyond metaplasia: unraveling the complex pathogenesis of autoimmune atrophic gastritis and its implications for gastric cancer risk. QJM.

